# Onset of persistent pseudomonas aeruginosa infection in children with cystic fibrosis with interval censored data

**DOI:** 10.1186/s12874-016-0220-5

**Published:** 2016-09-17

**Authors:** Wenjie Wang, Ming-Hui Chen, Sy Han Chiou, Hui-Chuan Lai, Xiaojing Wang, Jun Yan, Zhumin Zhang

**Affiliations:** 1 Department of Statistics, University of Connecticut, 215 Glenbrook Road, Storrs, 06269 CT USA; 2 Department of Biostatistics, Harvard T. H. Chan School of Public Health, 677 Huntington Ave, Boston, 02115 MA USA; 3 Department of Nutritional Sciences, University of Wisconsin, 1415 Linden Drive, Madison, 53706 WI USA; 4 Google, 76 Ninth Avenue, New York, 10011 NY USA; 5 Institute for Public Health Research, University of Connecticut Health Center, 195 Farmington Avenue, Farmington, 06032 CT USA

**Keywords:** Cox model, Dynamic model, Reversible jump Markov chain Monte Carlo, Time-varying effect

## Abstract

**Background:**

Persistent Pseudomonas aeruginosa (PPA) infection promotes lung function deterioration in children with cystic fibrosis (CF). Although early CF diagnosis through newborn screening (NBS) has been shown to provide nutritional/growth benefit, it is unclear whether NBS lowers the risk of PPA infection and how the effect of NBS vary with age. Modeling the onset age of PPA infection is challenging because 1) the onset age of PPA infection is interval censored in patient registry data; and 2) some risk factors such as NBS may have time-varying effects.

**Methods:**

This problem fits into the framework of a recently developed Bayesian dynamic Cox model for interval censored data, where each regression coefficient is allowed to be time-varying to an extent determined by the data.

**Results:**

Application of the methodology to data from the CF Foundation Patient Registry revealed interesting findings. Compared with patients with meconium ileus or diagnosed through signs or symptoms, patients diagnosed through NBS had significantly lower risks of acquiring PPA infection between age 1 and 2 years, and the benefit in survival rate was found to last up to age 4 years. Two cohorts of five years apart were compared. Patients born in cohort 2003–2004 had significantly lower risks of the PPA infections at any age up to 4 years than those born in 1998–1999.

**Conclusions:**

The study supports benefits of NBS on PPA infection in early childhood. In addition, our analyses demonstrate that patients in the more recent cohort had significantly lower risks of acquiring PPA infection up to age 4 years, which suggests improved CF treatment and care over time.

**Electronic supplementary material:**

The online version of this article (doi:10.1186/s12874-016-0220-5) contains supplementary material, which is available to authorized users.

## Background

Cystic fibrosis (CF) is a potentially lethal, lifelong recessive genetic disorder found mostly among Caucasians, affecting over 30,000 people in the United States [1]. It is caused by mutations in the gene for the cystic fibrosis transmembrane conductance regular protein. Chronic lung infections and obstructive lung diseases, eventually leading to cardiorespiratory failure, are the main causes of death (80 %) in patients with CF. *Pseudomonas aeruginosa* (PA), a ubiquitous environmental bacterium, is the most significant and prevalent pathogen that accelerates lung infections and shortens survival time of CF patients (e.g., [2, 3]). With improved treatment of CF, survival has increased significantly over time in the last three decades, with median predicted survival age increased from ∼28 years up to 40.7 years [1]. Early diagnosis of CF through newborn screening (NBS) provides long-lasting nutritional/growth benefits (e.g., [4, 5]). Nonetheless, findings of NBS on PA infections are inconsistent, possibly due to variable PA status (i.e., first, ever, current, persistent/chronic, or mucoid) and different statistical models used. PA infections can be transient or intermittent, especially using upper respiratory tract cultures in early childhood [6]. The first PA infection is most likely transient and, hence, is not a good indicator of lower airway infections. Transient PA infections can be eradicated by antibiotics treatments when they are diagnosed, but such eradication is difficult if they began early in life and became persistent [7]. On the other hand, as a primary cause of increased CF morbidity and mortality, persistent PA (PPA) infection can be used as a surrogate endpoint for survival [8]. Therefore, it is very important to characterize PPA infection in CF patients for treatment devise and patient management.

The Cystic Fibrosis Foundation (CFF) consensus report recommended that respiratory tract cultures should be obtained every three months in patients with stable pulmonary status [9]. In reality, however, the interval varies from days to months or years. Consequently, the onset age of PPA infection is interval censored. Two challenges are present in analyzing such data. First, standard Cox proportional hazards models for right censored data need to be adapted to account for the interval censoring scheme (e.g., [10–12]). Second, the effects of risk factors may be time-varying; for example, risks of PPA infection among different patient groups may be changing instead of fixed over time [13]. To address these challenges, a Cox model with time-varying coefficients for interval censored data is required.

We present a case study of analyzing the onset age of PPA infection using a dynamic Bayesian Cox model with time-varying coefficients for interval censored data [14]. Cox models with time-varying coefficients have been studied for interval censored data (e.g., [15–18]); a recent comprehensive treatment is [19]. Nonetheless, the dynamic Bayesian Cox model has a unique feature: it characterizes each coefficient by piecewise constant but the number of pieces is determined dynamically by the data instead of fixed. That is, the extent to which each covariate effect varies over time is driven by the need from the data. Some coefficients can be more time-varying while others can be less time-varying or approximately constant over time. The model is fitted in the Bayesian framework with reversible jump Markov chain Monte Carlo, and comparison to fully time-varying coefficient models [16] and standard Cox models are made with a Bayesian model selection criterion. Implementation of the methodology is publicly available in an open source R package dynsurv [20], which facilitates application to similar problems, especially analyses of interval censored event times from disease registry.

The case study revealed interesting findings that may not be obtained from standard techniques with time-independent covariate effects. Several factors that might be associated with onset of PPA infection in children with CF are examined, including gender, CF diagnostic modes, genotype, and birth cohort. Patients with pancreatic sufficiency (10.6 % of the total), who in general have milder CF were excluded from the analysis, and only those classical CF cases with pancreatic insufficiency were included. We hypothesized that children with CF in the more recent cohort, diagnosed earlier through NBS, or with mild genotypes were less likely to acquire PPA infections. The standard Cox model and its extensions allowing time-varying coefficients were fitted to test the hypothesis. The standard Cox model cannot capture how the effects vary over time. The dynamic Bayesian Cox model was found to outperform its competitors, uncovering the temporal dynamics of these effects. Our results suggested that patients diagnosed through NBS had significantly lower risk of PPA infection between age 1 to 2 years and the benefit in survival curve persisted up to age four years; patients born more recently were found to have lower risks of PPA infection up to age four years, which has not been reported before; no significant difference was found between female and male patients anywhere in the first four years.

## Methods

### Data

The study population consisted of patients reported in the 2008 CFF Patient Registry (CFFPR). CFFPR is a database established and managed by the CFF that tracks the health and treatments of people with CF in the US, collecting data for appropriately 28,000 patients annually [1]. Widely regarded as the nation’s only comprehensive source of validated data for CF, it provides clinicians and researchers access to a large sample of data that can be used to identify and study health trends, learn about effective treatments, and design clinical trials for potential new therapies (e.g., [21]). Patients with pancreatic insufficiency (receiving pancreatic enzymes replace therapies), who were genotyped and diagnosed before age 5 years in two birth cohorts (born in 1998–1999 denoted as BC[98–99], and born in 2003–2004 denoted as BC[03–04]) were selected from the 2008 CFFPR. A very small portion (0.68 %) of the patients who died before age 5 were not included. The remaining 2341 patients were included in our analyses and their followup data to the end of 2008 were extracted from the 2008 CFFPR for this study with appropriate administrative permissions.

We defined PA infection to be persistent if two or more positive PA infections occur within a 4–9 month time period without any negative results in between [22, 23]. The onset ages of PPA infection are subject to interval censoring. A PPA infection event is indicated by the first occurrence of consecutive positive PA infections in a 4–9 months period. In this case, the onset age of PPA infection is interval censored: the left endpoint is zero or the age at the last visit before the sequence, the right end point is the age at the first visit of the sequence. For example, a patient had the first two consecutive positive PA infections at age 2.34 and 2.93 years, respectively, and the last visit before the pair was at age 1.40 years. Then the censoring interval for this patient was (1.40, 2.34). If PPA infection was never identified for a patient within the observed followup period, the censoring interval was constructed from the age at the last visit to infinite (or right-censored). The stringent requirement to confirm for persistency in PA infections resulted 267 patients that were interval censored, with a median interval length of 0.30 year, and 2,074 patients that were right censored. Note that, although a child only enters the CFFPR after being diagnosed as having CF, the onset age of PPA infection can be either after or before the diagnosis age; in the latter case, the censoring interval would have left end point zero.

In addition to birth cohort, other risk factors including gender, mode of diagnosis, and genotype were also examined. Mode of CF diagnosis (DX) indicates how each patient in the CFFPR was diagnosed as having CF. Classified according to common clinical practices, DX is a categorical variable with four levels: (1) patients identified at birth because of an intestinal obstruction known as meconium ileus (MI); (2) patients diagnosed through NBS, typically in the neonatal period and often pre-symptomatic; (3) patients identified at variable ages because of positive family history (FH); and (4) patients identified because of symptoms (SYMP) other than MI at a median age of 8–9 months [24]. In general, most CF patients with pancreatic insufficiency will be diagnosed before age 5 [25]. The SYMP group does not necessarily include more severe patients with CF than the NBS group. All patients with CF regardless of diagnosis modes received similar standard cares after CF diagnosis. The potential pulmonary benefit of early diagnosis of pre-symptomatic patients through NBS has been supported by other studies (e.g., [13, 26]). Genotype (Geno) is classified based on the most common mutation F508del (e.g., [27, 28]) with three levels: (1) F508del homozygous — F508del/F508del (FF); (2) F508del heterozygous — F508del/other (FO); and (3) other/other (OO).

Tables 1 and 2 summarize the frequencies of two clinical variables DX and Geno by two demographic variables gender and BC. There were 1266 (54.1 *%*) patients in BC[98–99] and 1075 (45.9 *%*) patients in BC[03–04]. The two genders are more or less balanced. In the four DX groups, the SYMP group is the largest, and the FH group is the smallest; the NBS group has an increased relative frequency in BC[03–04] because more states in the US implemented NBS. In the three genotype groups, FF and FO consist of the majority of patients with CF, as about 90.6 % of patients with CF have at least one copy of the F508del mutation.

**Table 1:** Frequency table (with column percentage) of birth cohort, gender, mode of diagnosis and genotype

		BC[98–99]			BC[03–04]		
		Female	Male	Total	Female	Male	Total
DX	SYMP	385	341	726	249	281	530
		(58.1)	(56.6)	(57.4)	(48.9)	(49.7)	(49.3)
	MI	184	172	356	148	137	285
		(27.7)	(28.5)	(28.1)	(29.1)	(24.2)	(26.5)
	NBS	63	60	123	87	111	198
		(9.5)	(9.9)	(9.7)	(17.1)	(19.6)	(18.4)
	FH	31	30	61	25	37	62
		(4.7)	(5.0)	(4.8)	(4.9)	(6.5)	(5.8)
Geno	FF	357	319	676	276	311	587
		(53.8)	(52.9)	(53.4)	(54.2)	(54.9)	(54.6)
	FO	250	218	468	184	206	390
		(37.7)	(36.2)	(37.0)	(36.2)	(36.4)	(36.3)
	OO	56	66	122	49	49	98
		(8.5)	(10.9)	(9.6)	(9.6)	(8.7)	(9.1)

**Table 2:** Frequency table (with column percentage) of gender, genotype and mode of diagnosis

		Female				Male			
Geno		FF	FO	OO	Total	FF	FO	OO	Total
DX	SYMP	323	241	70	634	319	235	68	622
		(51.0)	(55.5)	(66.7)	(54.1)	(50.6)	(55.4)	(59.1)	(53.2)
	MI	197	120	15	332	176	100	33	309
		(31.1)	(27.6)	(14.3)	(28.3)	(27.9)	(23.6)	(28.7)	(26.4)
	NBS	82	54	14	150	98	66	7	171
		(13.0)	(12.4)	(13.3)	(12.8)	(15.6)	(15.6)	(6.1)	(14.6)
	FH	31	19	6	56	37	23	7	67
		(4.9)	(4.4)	(5.7)	(4.8)	(5.9)	(5.4)	(6.1)	(5.7)

### Preliminary analysis

As an exploratory analysis, the standard Cox model with constant coefficient for interval censored data was fitted to examine the association between the covariates and the onset age of PPA infection. Two methods are used: the iterative convex minorant (ICM) algorithm for interval censored data [12] implemented in R package intcox; and the Bayesian method implemented in R package dynsurv. The levels Female, SYMP, FF and BC[98–99] were, respectively, used as the reference levels for gender, mode of diagnosis, genotype and birth cohort in hereafter model fitting Table 3 summarizes the estimated coefficients. As package intcox does not provide standard errors for the parameter estimates, they were obtained from 1,000 bootstrap samples. The results from the Bayesian inference were obtained with the default prior choices in package dynsurv.

**Table 3:** Estimated coefficient by iterated convex minorant (ICM) algorithm and the Bayesian posterior mean in standard Cox model for onset age of PPA infection

	ICM (intcox)			Bayesian (dynsurv)	
	Estimate	Std. Err.	Pr(>|*z*|)	Estimate	95 % credible interval
Gender (Male)	0.158	0.121	0.191	0.128	(−0.111, 0.360)
DX (MI)	−0.048	0.142	0.736	−0.068	(−0.347, 0.208)
DX (NBS)	−0.435	0.221	0.048	−0.422	(−0.857,−0.028)
DX (FH)	−0.030	0.283	0.917	−0.011	(−0.537, 0.509)
Geno (FO)	−0.137	0.139	0.327	−0.139	(−0.411, 0.123)
Geno (OO)	0.200	0.198	0.312	0.158	(−0.255, 0.534)
BC[03-04]	−0.450	0.130	0.001	−0.497	(−0.751,−0.242)

The estimates of the time-independent coefficients from the two methods are reasonably close, leading to similar observations. Patients diagnosed through NBS had significantly lower risks at level 5 % compared with those diagnosed by SYMP; patients in BC[03–04] had significantly lower risks than those in BC[98–99] at the 5 % level for PPA infection. Nonetheless, the standard Cox model cannot capture any potential temporal dynamics of covariate influences which have been reported for PA infections [13]. We therefore fitted the Bayesian dynamic Cox model with data driven time-varying regression coefficients [14].

### Bayesian dynamic cox model

#### Model and likelihood

Suppose that *n* independent subjects are observed. For subject *i*,*i*=1,…,*n*, let *T*_*i*_ be the unobserved event time of interest (onset age of PPA infection), and (*L*_*i*_,*R*_*i*_] be the observed censoring interval containing *T*_*i*_. Let **X**_*i*_ be a *p*-dimensional vector of covariates for subject *i*. To go beyond the proportional hazards assumption in the standard Cox model, the dynamic Cox model [14] allows the covariate coefficient to be time-varying:

1 $$ \lambda(t|{\mathbf{X}}_{i}) = \lambda_{0}(t)\exp\left\{{\mathbf{X}}_{i}^{\top} \boldsymbol{\beta}(t)\right\},  $$

where *λ*_0_(*t*) is the baseline hazard and ***β***(*t*) is the *p*-dimensional regression coefficients of **X**_*i*_ at time *t*. Model (1) is seemingly the same as the time-varying coefficient Cox model [16]. Both models assume that *λ*_0_(*t*) and ***β***(*t*) are left continuous step functions and the potential jump points are limited to a fine grid of time points *G*={0=*s*_0_<*s*_1_<…<*s*_*K*_<*∞*}. Sinha et al. [16] place the jump points at all *K* grid points, which is unnecessary for coefficients that are relatively stable. This motivated Wang et al. [14] to allow the number of jump points *J*, *J*≤*K*, to be covariate specific and data-driven; some coefficients can be more time-varying than others.

A data augmentation approach facilitates the inferences. For a finite censoring interval (*R*_*i*_<*∞*), let d*N*_*i*,*k*_=*I*(*T*_*i*_∈(*s*_*k*−1_,*s*_*k*_]), indicating whether *T*_*i*_ is in the *k*th interval on the grid. The at risk indicator *Y*_*i*,*k*_ is determined by d*N*_*i*,*k*_’s. If d*N*_*i*,*k*_=1 for certain *k*, then *Y*_*i*,*l*_=1 for *l*<*k*,*Y*_*i*,*l*_=0 for *l*>*k* and *Y*_*i*,*k*_=(*T*_*i*_−*s*_*k*−1_)/*Δ*_*k*_, where *Δ*_*k*_=*s*_*k*_−*s*_*k*−1_ is the width of the *k*th interval. For a right censoring interval (*R*_*i*_=*∞*),d*N*_*i*,*k*_=0 for all *k*, and *Y*_*i*,*k*_=*I*(*s*_*k*_≤*L*_*i*_). The information in *T*_*i*_ is now equivalently contained in $\{{\mathrm {d}} N_{i,k}, Y_{i,k}\}_{k=1}^{K}$, which are treated as missing data. Let ***Θ***={log*λ*_0_(*t*),***β***(*t*); *t*>0} contain all the piecewise constant parameters of the baseline hazard and the regression coefficients. When the event indicator d*N*_*i*,*k*_ and at-risk indicator *Y*_*i*,*k*_,*k*=1,…,*K*, are all observed, the complete data likelihood is

$$\begin{array}{*{20}l} &{} L\left(\boldsymbol{\Theta}|\left\{{\mathrm{d}} N_{i,k}, Y_{i,k}\}^{K}_{k=1}, \mathbf{X}_{i}; i = 1, \ldots, n \right\} \right) \\ {}= &\!\prod_{i=1}^{n}\prod_{k=1}^{K}\! \left\{\lambda_{k} \exp(\mathbf{X}_{i}^{T}\boldsymbol{\beta}_{k})\right\}^{{\mathrm{d}} N_{i,k}} \!\exp\! \left\{\,-\,\Delta_{k}\lambda_{k} \!\exp(\mathbf{X}_{i}^{T}\boldsymbol{\beta}_{k})\!Y_{i,k}\!\right\}\!. \end{array} $$

#### Prior specification

As log*λ*_0_(*t*) can be viewed as the regression coefficient of ones, its prior is specified similar to other component in ***Θ***(*t*). Without loss of generality, let *θ*(*t*) be a component in ***Θ***(*t*). The prior distribution of *J* is discrete uniform over {1,…,*K*}. Given that there are *J* jumps in *θ*(*t*), the jump times 0<*τ*_1_<…<*τ*_*J*_=*s*_*K*_ are random except the last one. Given both *J* and the jump times, a hierarchical Markov process prior is specified for *θ*(*t*):

$$\begin{array}{*{20}l} \theta(\tau_{1})|\omega &\sim {\mathcal{N}}(0, a_{0}\omega), \qquad a_{0} > 0, \\ \theta(\tau_{j})|\theta(\tau_{j-1}), \omega &\sim {\mathcal{N}}(\theta(\tau_{j-1}), \omega), \qquad j = 2, 3, \ldots, J,\\ \omega & \sim \mathcal{IG}(\alpha_{0}, \xi_{0}), \qquad \alpha_{0} > 0,\ \xi_{0} > 0, \end{array} $$

where *a*_0_,*α*_0_, and *ξ*_0_ are hyperparameters, $\mathcal {N}\left (\mu, \sigma ^{2}\right)$ is a normal distribution with mean *μ* and variance *σ*^2^, and $\mathcal {IG}(\alpha _{0}, \xi _{0})$ is an inverse gamma distribution with shape parameter *α*_0_ and scale parameter *ξ*_0_ such that the mean is *ξ*_0_/(*α*_0_−1) for *α*_0_≤1. The introduction of *ω* gives much more room to adjust the amount of penalty on the smoothness of *θ*(*t*) automatically than the case where *ω* is specified as a hyperparameter [16]. The prior on the variance of *θ*(*τ*_1_) is specified to be more noninformative by multiplying a hyperparameter *a*_0_>1.

Each component in ***Θ*** has its own *J* and *ω*.

#### Posterior computation

A reversible jump Markov chain Monte Carlo (RJMCMC) algorithm [29] is necessary for posterior sampling to make inferences because the dimension *J* of each component in ***Θ*** is dynamic. Let d***N***={d*N*_*i*,*k*_:*i*=1,…,*n*, *k*=1,…,*K*} and ***Y***={*Y*_*i*,*k*_:*i*=1,…,*n*, *k*=1,…,*K*}. In addition to the parameters of interest ***Θ***, the augmented event indicators and at-risk indicators for finite censored intervals and the second level parameters ***ω***={*ω*_0_,*ω*_1_,…,*ω*_*p*_}, which correspond to ***Θ***={*Θ*_0_,*Θ*_1_,…,*Θ*_*p*_} also need to be updated in the iterations. A Gibbs sampling framework draws {*T*_*i*_:*R*_*i*_<*∞*},***Θ*** and *ω*’s iteratively as follows:

1. For each subject *i* with *R*_*i*_<*∞*, sample event time *T*_*i*_ given ***Θ***, and compute event indicators d*N*_*i*,*k*_ and at-risk indicators *Y*_*i*,*k*_,*k*=1,…,*K*.
2. For each *j*∈{0,1,…,*p*}, sample *Θ*_*j*_ given *Θ*_−*j*_ (all components in ***Θ*** except the *j*th), d***N***,***Y***, and ***ω***.
3. For each *j*∈{0,1,…,*p*}, sample *ω*_*j*_ given ***Θ*** from an $\mathcal {IG}$ distribution resulting from the conjugate prior of *ω*_*j*_.

The second step is where the reversible jump part comes in, and a random number of jumps *J*_*i*_ for each *i*=0,1,…,*p* leads to a posterior with variable dimensions. Three types of moves — birth, death, and update — with probability 0.35,0.35 and 0.3, respectively, are used to add a jump point, remove a jump point, and update the jump sizes with no jump points fixed. See [14] for details.

It is often of interest to compare the survival curves of two groups of subjects. This was not discussed in [14] but can be conveniently constructed from the posterior sample. Given covariate vector **X**, the survival function *S*(*t*|**X**) corresponding to the piecewise constant hazard *λ*(*t*|**X**) in Model (1) evaluated at a grid point *s*_*k*_,*k*=1,…,*K*, is

$$\begin{array}{*{20}l} S(s_{k} |{\mathbf{X}}, \boldsymbol{\Theta}) = \exp\left\{-\sum_{i \le k} \lambda_{i} (s_{i}) e^{{\mathbf{X}}^{\top} \boldsymbol{\beta}(t)} \right\}, \end{array} $$

Let ***Θ***^(*i*)^ be the posterior draw of ***Θ*** from the *i*th RJMCMC iteration, *i*=1,…,*N*. The survival function at each grid point *s*_*k*_,*k*=1,…,*K*, is estimated by the posterior mean

$$\begin{array}{*{20}l} \hat{S}(s_{k} | {\mathbf{X}}) = \frac{1}{N}\sum_{i=1}^{N} S(s_{k}|{\mathbf{X}}, \boldsymbol{\Theta}^{(i)}). \end{array} $$

The credible interval for the survival curve at *s*_*k*_ can be constructed based on the quantiles of *S*(*s*_*k*_|**X**,***Θ***^(*i*)^),*i*=1,…,*N*. For two different sets of covariates **X**_1_ and **X**_2_, the difference in survival curves at *s*_*k*_ can be estimated $\hat {S}(s_{k} | {\mathbf {X}}_{1}) - \hat {S} (s_{k} | {\mathbf {X}}_{2})$, with credible intervals constructed from the quantiles of *S*(*s*_*k*_|**X**_1_,***Θ***^(*i*)^)−*S*(*s*_*k*_|**X**_2_,***Θ***^(*i*)^),*i*=1,…,*N*.

#### Convergence check and model comparison

Convergence check for the RJMCMC is challenging. The parameters ***Θ***(*t*) as functions of time retain their interpretations when the sampler moves across models with different dimensions, and are to be monitored [30]. Nonetheless, each component in ***Θ***(*t*) has *K* points, resulting *K*(*p*+1) parameters which are too many to monitor altogether. Posterior samples of each component in ***Θ***(*t*) as a curve can be made into animations for visual checking. Alternatively, the curves evaluated at a small number of fixed time points can be monitored with the usual convergence checks. The number of pieces *J* for each curve is more difficult to converge than points on the curves from our experience, so it provides an easy alternative to monitor.

The advantage of the dynamic model in comparison to the standard Cox model and the fully time-varying coefficient Cox model [16] can be shown through model comparison in the Bayesian framework. Due to random dimension of the dynamic model, Wang et al. [14] recommended to use the log pseudo marginal likelihood (LPML). For a model ${\mathcal {M}}$, the LPML is

$$\text{LPML}_{{\mathcal{M}}} = \Sigma^{n}_{i=1}\log \left[\text{CPO}_{{\mathcal{M}}}(i)\right], $$

where the CPO represents the conditional predictive ordinate, which is essentially a Bayesian cross-validation approach [31]. In the current application, for the *i*th subject, the CPO statistics is defined as

$$\text{CPO}_{{\mathcal{M}}}(i) = \Pr\left(T_{i} \in [L_{i}, R_{i}]|\mathbf{D}_{obs}^{(-i)}\right), $$

where $\mathbf {D}_{obs}^{(-i)}$ is the observed interval censored data with the *i*th subject removed. In practice, $\text {CPO}_{{\mathcal {M}}}(i)$ can be calculated as the harmonic mean of copies of Pr(*T*_*i*_∈(*L*_*i*_,*R*_*i*_]∣***Θ***,**X**_*i*_) evaluated at RJMCMC samples from ***Θ*** given the observed data. Model with higher LPML are preferred to models with lower LPML.

## Results

To make a fair comparison between the two cohorts, we excluded data after age 5 years from patients in BC[98–99] because patients in BC[03–04] had no data after age 5 years reported in the 2008 CFFPR. The time grid was set to be equally spaced in (0,5) with increment 0.1, which is sufficiently fine to capture the temporal dynamics of the covariate effects [13] and at the same time not too dense to cause unnecessarily large computing burden. To conform with the grid, the end points of the censoring interval were rounded: the left end was rounded down, and the right end was rounded up to the nearest 0.1 year. The age window we report for the PPA infection, however, was chosen to be (0,4) years, because at the data extraction time (end of 2008), patients born in 2004 did not reach 5 years old yet, and the definition of PPA involves at least two visits of 4–9 months apart.

The prior for the regression coefficients *Θ*(*t*) was set as the Markov process prior as described earlier. The multiplier *a*_0_ was fixed at 100 to allow for a noninformative specification for the first piece of coefficient function. The prior distribution of *ω* was set to be $\mathcal {IG}(1, 1)$, which is quite vague as it does not even have finite first moment. Alternative priors were used for sensitivity analysis later. With R package dynsurv [20], 200,000 RJMCMC iterations were generated. With a burn-in period of 130,000, the remaining iterations were thinned by 10, resulting in a sample of size 7,000. This sample was checked for convergence and used for computing the LPML for model comparisons with competing models. The trace plots of *J* for all coefficients are presented in Additional file 1.

**Fig. 1:**
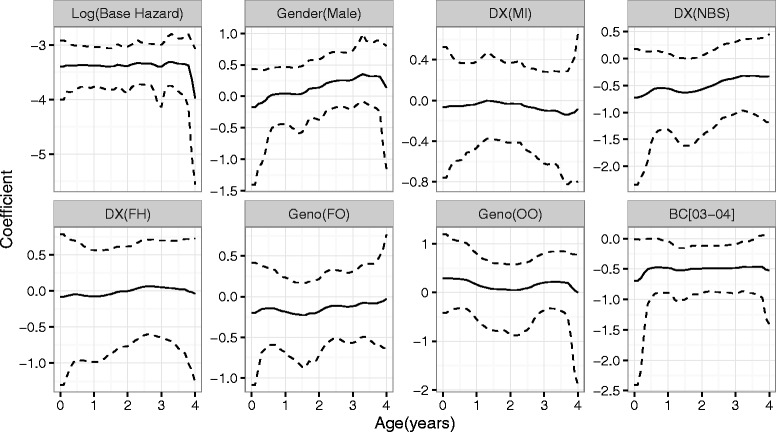
Estimates of coefficient function (*solid* line) and 95 % credible intervals (*dash* line) from dynamic model for the onset age of PPA infection

The estimated time-varying coefficients with their 95 *%* credible intervals from the dynamic Cox model (1) for the onset age of PPA infection are displayed in Fig. 1. The temporal dynamics of all the coefficients revealed subtle, interesting findings that cannot be seen from the standard Cox models with time-independent coefficients reported in Table 3. Males appeared to have higher risks of acquiring PPA infection than females after age 2, but the effect was not significant at 5 *%* at any time before age 4. Patients diagnosed through NBS had lower risk of PPA infection than those diagnosed through SYMP, and the difference was significant at 5 % level between age 1 and 2 years; afterwards, the effect diminished. Patients diagnosed by MI or FH had no difference in PPA infection risk in comparison to those diagnosed by SYMP, with their effects quite flat around zero at all ages before 4 years. Similarly, patients with genotype FO or OO had no significant difference in PPA risk in comparison with those with genotype FF at any time before age 4 years either. Patients in BC[03–04] had significantly and persistently lower risk of PPA infection than those in BC[98–99] at all ages before 4 years.

**Fig. 2:**
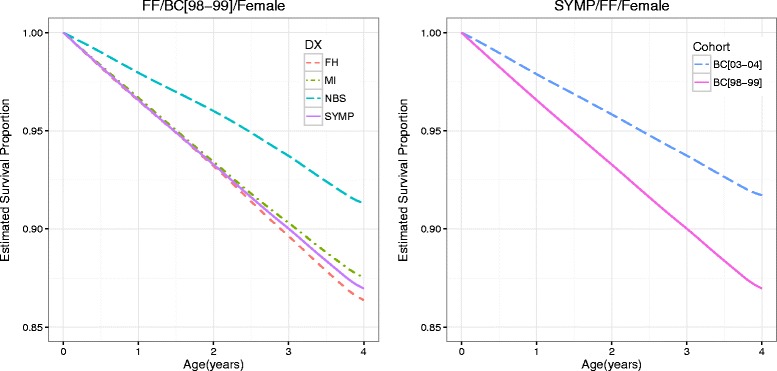
Estimated survival curves of patients from different treatment groups or cohorts. The *left* panel shows the estimated survival function of females with genotype FF from cohort BC[98–99] in different DX groups. The *right* panel shows the estiamted survival function of females with genotype FF diagnosed via symptoms from different birth cohorts

It is of interest to compare the survival curves between subgroups of CF patients, which may provide additional clinical insights to those obtained from instantaneous risk modeled in (1). Figure 2 shows the estimated survival curves of patient groups with certain covariate information. The left panel compares the survival curves of four female patient groups with genotype FF in BC[98–99], each from one of the four diagnosis modes: SYMP, NBS, MI, and FH. Females diagnosed through NBS had apparently longer survival time to PPA infection than those in the other three groups, whose survival curves were very close to one another. The right panel compares the survival curves of two female patient groups with genotype FF and diagnosed through SYMP, one in BC[03–04] and the other in BC[98–99]. Females in BC[03–04] has longer survival time to PPA infection than those in BC[98–99].

**Fig. 3:**
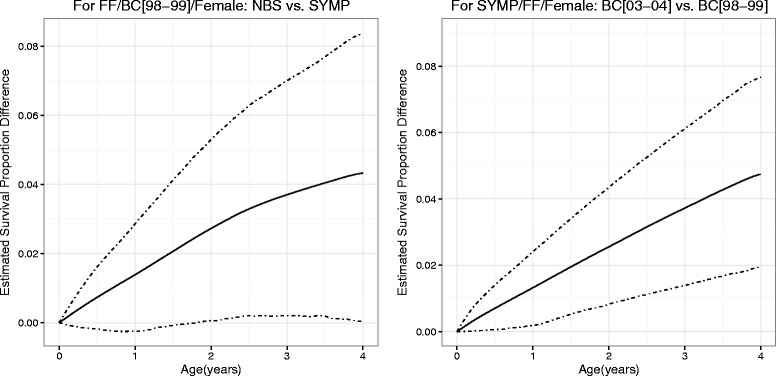
Estimated differences between survival functions (*solid* line) for different patient groups and their 95 % credible intervals (*dash-dot* line)

The differences between the survival curves for subgroups of interests and their 95 % credible intervals are displayed in Fig. 3. The left panel shows the difference between females diagnosed through NBS and those diagnosed through SYMP, both with genotype FF from BC[98–99]. The largest difference was about 4.4 % at age 4 years. The 95 % credible intervals barely covered zero before age 2 years and excluded zero between age 2 and 4 years. This suggest that, although the difference in instantaneous risk of PPA infection between patients diagnosed by NBS and those diagnosed by SYMP became insignificant after age 2 (Fig. 1), the benefit of NBS in lowering PPA infection risk sustained to at least age 4 years in children CF patients. The right panel shows the difference in survival curves between females from BC[03–04] and females from BC[98–99], both with genotype FF and diagnosed through SYMP. The credible intervals were above zero during age 0 to 4. Females in BC[03–04] had a significantly greater survival rate to PPA infection than those females in BC[98–99] at the 5 % level. The difference was almost linearly increasing and attained the highest point of about 5 % at age 4, which suggests significant improvements in CF patient care in the recent cohort that is not accounted for by the other predictors. Similar results were observed in other group comparisons (data not shown).

The performance of the dynamic Cox model (1) in comparison with competing models for the PPA analysis can be assessed using LPML. Let $\mathcal {M}_{1}, \mathcal {M}_{2}$, and $\mathcal {M}_{3}$ be, respectively, the standard Cox model with time-independent coefficients, the fully time-varying coefficient model of Sinha et al. [16], and model (1). Independent gamma priors with shape 0.1 and rate 0.1 were placed on the pieces of baseline hazards in both $\mathcal {M}_{1}$ and $\mathcal {M}_{2}$. For each time-independent regression coefficient in $\mathcal {M}_{1}$, an independent $\mathcal {N}(0, 1)$ prior was specified. An independent Markov process prior with a fixed *ω*=1 was placed on each time-varying coefficient in $\mathcal {M}_{2}$ [16]. The LPML values are −6275.93,−5995.99, and −1925.58 for $\mathcal {M}_{1}, \mathcal {M}_{2}$, and $\mathcal {M}_{3}$, respectively. Model $\mathcal {M}_{3}$ outperformed the other two by a drastic gain. Compared with $\mathcal {M}_{1}$, it allows temporal dynamics in regression coefficients that are not possible in $\mathcal {M}_{1}$. Although the coefficients in Fig. 1 look flat, a complete time-invariant coefficient Cox model would not capture the subtle dynamics, especially in the coefficients of gender and DX[NBS]. Compared with $\mathcal {M}_{2}$, it has a much smaller number of effective parameters and provides much narrower credible intervals for the time-varying coefficients (see plot for estimated coefficients from $\mathcal {M}_{2}$ in Additional file 1). Specifically, the posterior mean number of pieces *J* for the coefficients in $\mathcal {M}_{3}$ ranges from 2 to 5 (see the histograms in Additional file 1), which are much smaller than *K*=50, the unnecessarily large number of pieces in $\mathcal {M}_{2}$. The dynamic model $\mathcal {M}_{3}$ strikes a balance between flexibility and parsimony of the Cox model in this application. It is also a compromise between bias variance tradeoff.

Sensitivity analysis was performed with a few other specification of hyperparameters and the results were fairly stable. For example, when prior $\mathcal {IG}(\alpha _{0}, 1)$ was specified for *ω* with *α*_0_∈{0.5,2,3,4} under ${\mathcal {M}}_{3}$. The estimated coefficients from each prior specification are plotted in Web Figures in Additional file 1, which are virtually unchanged from Fig. 1. The dynamic model ${\mathcal {M}}_{3}$ still outperforms $\mathcal {M}_{1}$ and $\mathcal {M}_{2}$ by a large margin in LPML.

## Discussion

We examined risk factors associated with the onset age of PPA infection in children with CF, using the Bayesian dynamic Cox model, which enables us to deal with interval-censored data and capture the time-varying effects of risk factors. The results of the analyses generated interesting findings on PPA infections in young children with CF. The early benefit of NBS on PPA infection persisted until the end of our study at age 4 years, as shown from the estimated survival curves constructed from posterior samples, although no additional benefit in instantaneous risk was found during ages 2–4 years. Such time-varying effect of NBS cannot be observed from time-independent Cox model. This observation echoes findings regarding growth benefit of NBS: early growth benefit of NBS sustained through adolescence with no additional benefit observed during puberty in the Wisconsin Randomized Clinical Trial (RCT) of CF Neonatal Screening project [4, 5]. It is noted that after CF diagnosis, all children received standard cares regardless their diagnostic modes. These findings indicate that children diagnosed through conventional methods maintain a similar disease progression as children diagnosed through NBS did after diagnosis, with disease outcomes remaining below but neither falling further behind nor catching up appreciably. The results justify the importance of and the need for continuing efforts to improve CF care after NBS.

Nevertheless, concerns regarding earlier acquisition of PA in children diagnosed through NBS still exist, as the NBS arm of the Wisconsin RCT had higher rates of ever PA positive infections because of earlier exposure to older patients with CF until care protocols were modified to ensure segregated followup care [32]. Since that time, following the recommendations of the Centers for Disease Control and Prevention [33] to maintain PA-segregated care when implementing CF NBS, no evidence indicates that NBS is associated with early PA acquisition [34–37]. In fact, analysis utilizing older CFFPR cohort born 1986–2000 found that NBS results in lower prevalence of PA infection compared with traditional diagnosis via symptoms/signs in the first seven years of life [13]. Such benefit attenuated with age and became insignificant by age 10 years. The present study also demonstrated the time-varying effect of NBS on PPA in the early childhood. Further studies are needed to examine its long-term effect to adolescence, when lung function may start to decline and lung disease progressively deteriorates [38].

We used the birth cohort as a surrogate to capture all the effects that are not captured by gender, diagnosis mode, and genotype. Our analyses also demonstrate that patients born in cohort 2003–2004 had significantly lower risks of the PPA infections at any age up to 4 years than those born in 1998–1999. Since NBS was increasing implemented and became nationwide in the U.S in 2010, children in the recent cohort are more likely to be diagnosed earlier through NBS. After adjusting for diagnostic modes, however, cohort effect still exists, indicating significant advances in CF treatment over time. The effects of gender and genotype are generally flat and not significant at the 5 % level. Nonetheless, using F508del mutation only to define genotype had limitations, as some other CF-causing mutations are also associated with severe CF phenotype [39, 40]. As more mutations are studied for molecular defect consequences, our future analyses is to re-define genotype using mutation class information. It is worth pointing out that there is no standard definition for chronic/persistent PA. Other approaches to define PPA as well as mucoid PA will be explored in our future analyses. The different frequency in PA cultures can also influence the determination of the onset age. More frequent cultures would yield more accurate estimate. Given the visits occur fairly regularly (every three months), the influence of irregular cultures on the analyses would be small.

## Conclusions

The statistical methods that we used appropriately address a challenging feature of the CFFPA data — interval censored onset age of PPA infections. Moreover, our model allows the effects of the risk factors to be time-varying. Therefore, the findings using this new method are more convincing than those using the existing models based on less sophisticated statistical methodologies. Our study supports benefits of NBS on PPA infection in early childhood. In addition, patients in the more recent cohort were found to have significantly lower risks of acquiring PPA infection up to age 4 years, which suggests improved CF treatment and care over time.

## Additional file

Additional file 1 Model diagnosis. The additional file mainly includes diagnosis plots and sensitivity check for the dynamic Cox model. (PDF 338 kb)

## Abbreviations

CF: Cystic fibrosis
CFF: Cystic fibrosis foundation
CFFPR: CFF Patient Registry
NBS: newborn screening
PA: Pseudomonas aeruginosa
PPA: Persistent pseudomonas aeruginosa
RCT: Randomized clinical trial
RJMCMC: Reversible jump Markov chain Monte Carlo
