# Supplementary material for: Onset of persistent pseudomonas aeruginosa infection in children with cystic fibrosis with interval censored data
Source: BMC Med Res Methodol. 2016 Sep 17;16:122. doi: 10.1186/s12874-016-0220-5 (PMC5027124; doi:10.1186/s12874-016-0220-5)
Supplement: Additional file 1 — Model diagnosis. The additional file mainly includes diagnosis plots and sensitivity check for the dynamic Cox model. (PDF 338 kb) [file 12874_2016_220_MOESM1_ESM.pdf]

RESEARCH

Supplementary Materials for "Onset of Persistent Pseudomonas Aeruginosa Infection in Children with Cystic Fibrosis with Interval Censored Data"

Wenjie Wang[1], Ming-Hui Chen[1], Sy Han Chiou[2], Hui-Chuan Lai[3], Xiaojing Wang[4], Jun Yan[1,5*] and Zhumin Zhang[3]

*Correspondence:
jun.yan@uconn.edu
[1]Department of Statistics, University of Connecticut, 215 Glenbrook Road, 06269 Storrs, USA
Full list of author information is available at the end of the article

Appendix A: Diagnosis Plots of the Dynamic Cox Model

In this section, we include diagnosis plots for the Bayesian dynamic Cox model [1] applied in the analysis of the PPA infection. The trace-plots of number of pieces J of each covariates, which is the most difficult to converge, are shown in Figure 1. The plots appear to be satisfactory.

In addition, the histogram shown in Figure 2 provides a graphic summaries of J for each coefficient in the dynamic Cox model. The mode number of pieces for each coefficient ranges from 1 (for Dx(MI)) to 4 (for logarithm of base hazard).

Appendix B: Sensitivity Check for the Dynamic Cox Model

The estimated coefficient function shown in Figure 3 to Figure 6 provides a visual comparison of results from the dynamic Cox model when different prior is specified for ω, the variance of the hierarchical Markov process for each coefficient. The scale parameter in $\mathcal{IG}$ prior was fixed at 1; the shape parameter was specified to be 0.5, 2, 3, and 4, respectively. The fitted dynamic coefficients are virtually the same as those from the $\mathcal{IG}(1, 1)$ prior.

Appendix C: Estimates from the Time-Varying Coefficient Cox Model

The estimated coefficient function from the time-varying coefficient Cox model [2] is shown in Figure 7. The results were obtained from package dynsurv with default prior choices.

Author details

[1]Department of Statistics, University of Connecticut, 215 Glenbrook Road, 06269 Storrs, USA. [2]Department of Biostatistics, Harvard T. H. Chan School of Public Health, 677 Huntington Ave, 02115 Cambridge, USA. [3]Department of Nutritional Sciences, University of Wisconsin, 1415 Linden Drive, 53706 Madison, USA. [4]Google, 76 Ninth Avenue, 10011 New York, USA. [5]Institute for Public Health Research, University of Connecticut Health Center, 195 Farmington Avenue, 06032 Farmington, USA.

References

1. Wang, X., Chen, M.-H., Yan, J.: Bayesian dynamic regression models for interval censored survival data with application to children dental health. Lifetime Data Analysis **19**(3), 297–316 (2013). doi:10.1007/s10985-013-9246-8
2. Sinha, D., Chen, M.-H., Ghosh, S.K.: Bayesian analysis and model selection for interval-censored survival data. Biometrics **55**(2), 585–590 (1999)

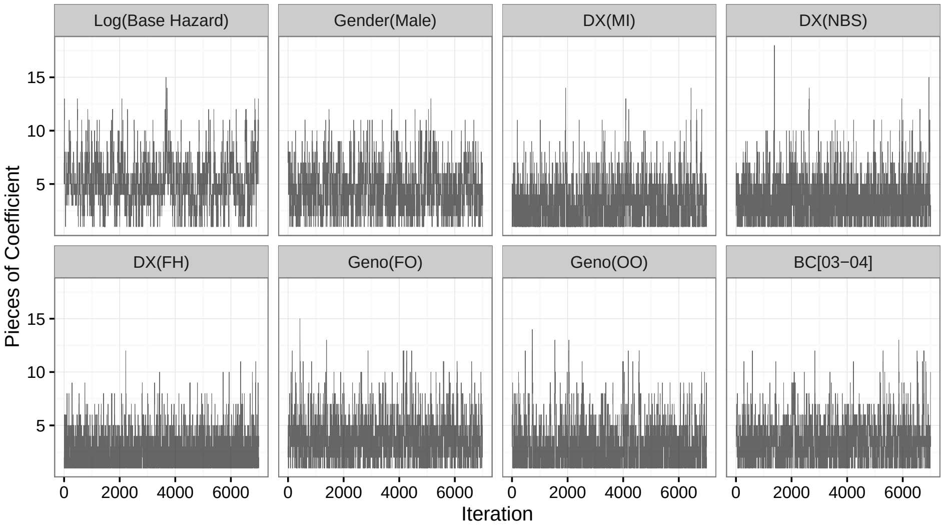

Figure 1 Trace-plots of number of pieces J of each covariate coefficient from the dynamic Cox model for the PPA infection.

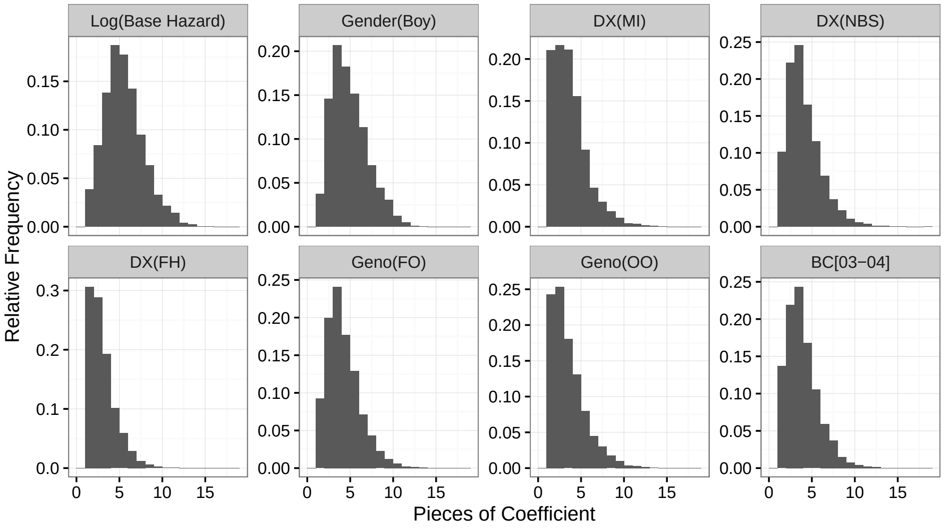

Figure 2 Histograms of number of pieces J for each covariate coefficient from the dynamic Cox model for the PPA infection.

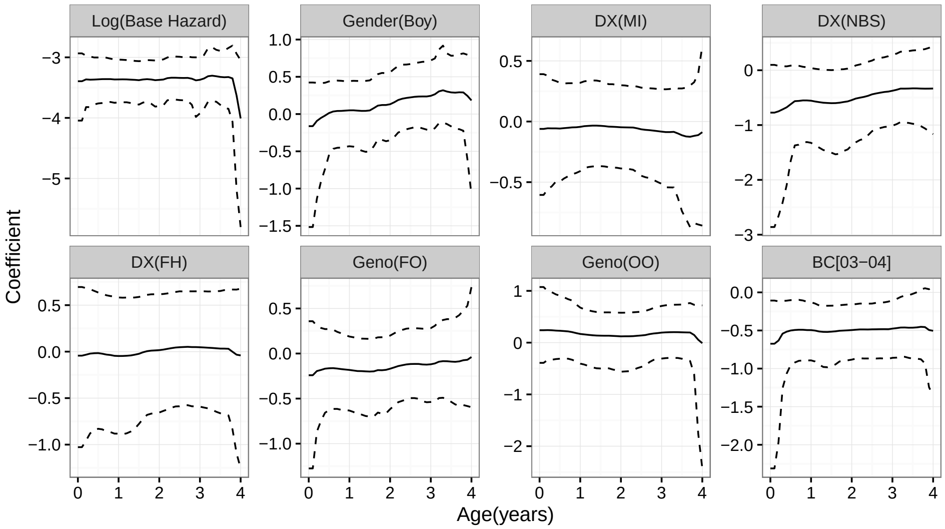

Figure 3 Estimates of coefficient function and 95% credible intervals from the dynamic Cox model under $\mathcal{IG}(0.5, 1)$ prior for ω.

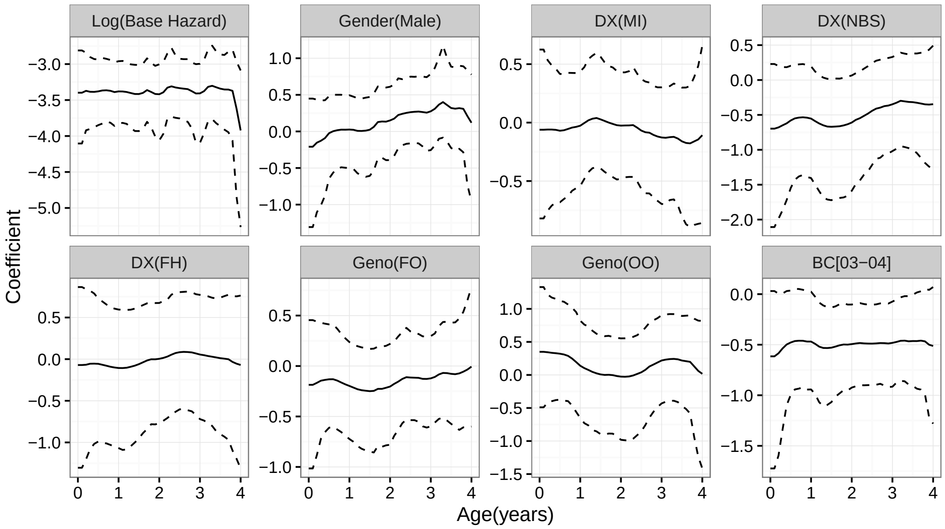

Figure 4 Estimates of coefficient function and 95% credible intervals from the dynamic Cox model under $\mathcal{IG}(2, 1)$ prior for ω.

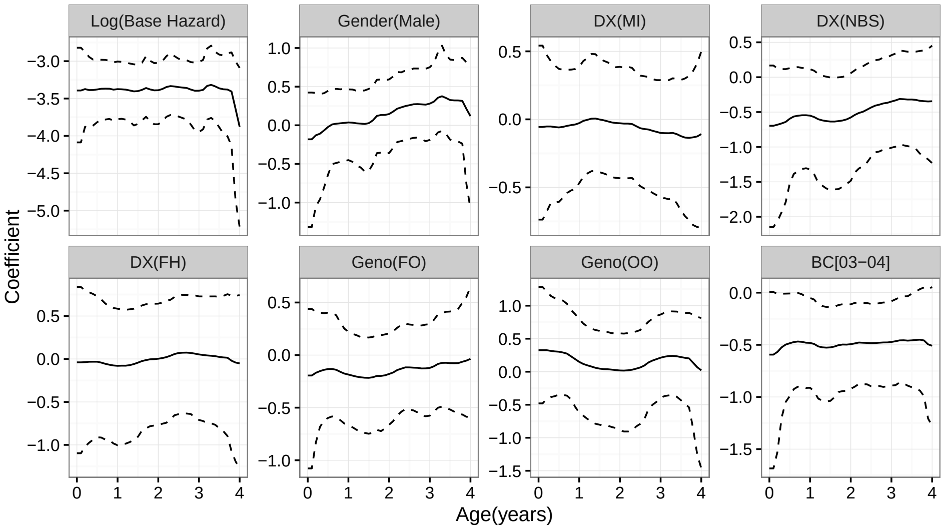

Figure 5 Estimates of coefficient function and 95% credible intervals from the dynamic Cox model under $\mathcal{IG}(3, 1)$ prior for ω.

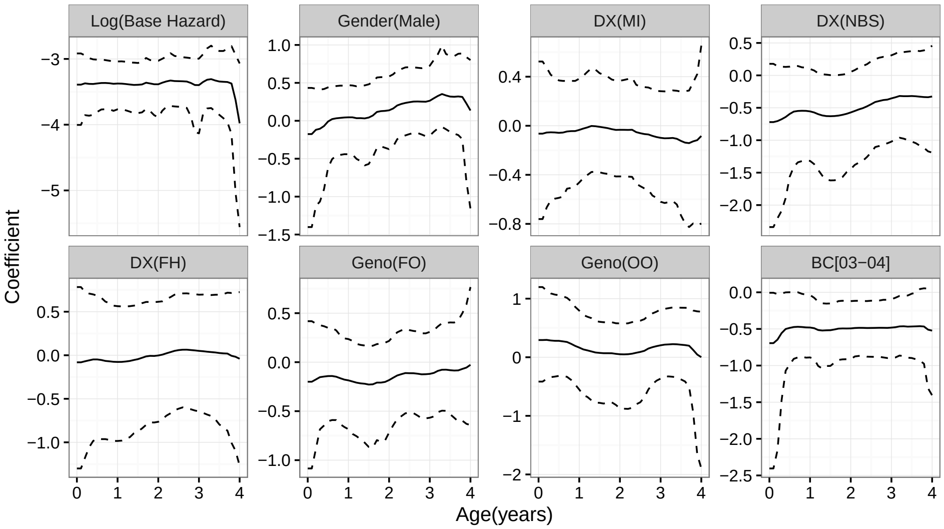

Figure 6 Estimates of coefficient function and 95% credible intervals from the dynamic Cox model under $\mathcal{IG}(4, 1)$ prior for ω.

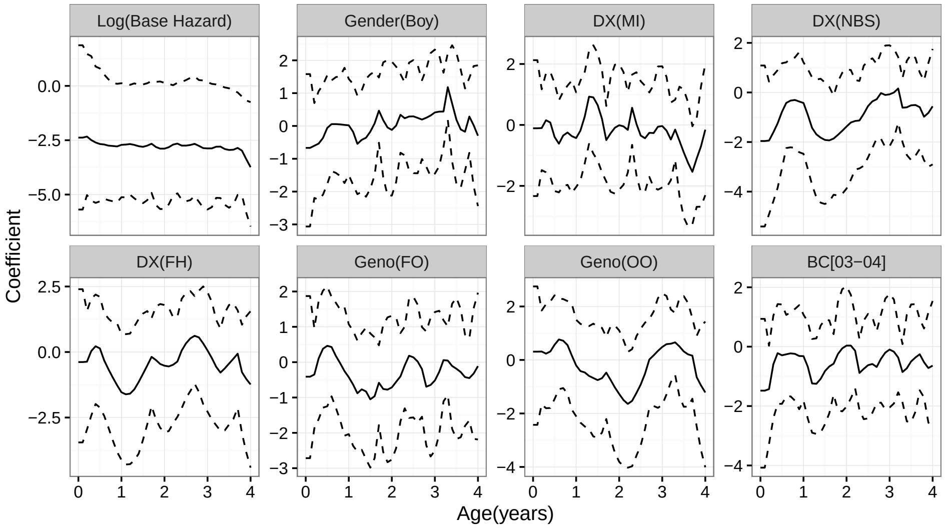

Figure 7 Estimates of coefficient function and 95% credible intervals from the time-varying coefficient Cox model.
